# Analysis of the communities of an urban mobile phone network

**DOI:** 10.1371/journal.pone.0174198

**Published:** 2017-03-23

**Authors:** Federico Botta, Charo I. del Genio

**Affiliations:** 1 Centre for Complexity Science, University of Warwick, Gibbet Hill Road, CV4 7AL, Coventry, United Kingdom; 2 School of Life Sciences, University of Warwick, Gibbet Hill Road, CV4 7AL, Coventry, United Kingdom; Universite de Namur, BELGIUM

## Abstract

Being able to characterise the patterns of communications between individuals across different time scales is of great importance in understanding people’s social interactions. Here, we present a detailed analysis of the community structure of the network of mobile phone calls in the metropolitan area of Milan revealing temporal patterns of communications between people. We show that circadian and weekly patterns can be found in the evolution of communities, presenting evidence that these cycles arise not only at the individual level but also at that of social groups. Our findings suggest that these trends are present across a range of time scales, from hours to days and weeks, and can be used to detect socially relevant events.

## 1 Introduction

The last decade has seen a deep change in the way scientists investigate and model social systems. The availability of data, generated through interactions with technological devices, allowed researchers to shift their focus, from qualitative to quantitative and computational studies of society [1, 2]. The increasing pervasiveness of always-on technology creates a vast amount of information that closely reflects human activity. This provides insight into the behaviour of people across the levels of their environment, from the individual scale, through groups and communities, to the global sphere, enabling the creation of models with predictive power [3–5]. Data recorded from mobile phones are a target of choice for research of this kind, offering a high granularity and being effectively ubiquitous in our society [6–12].

One of the main approaches to analyse this type of complex data is to model them as a network, i.e., a structure in which connections (edges) link pairs of discrete elements (nodes) [13–15]. In the case of mobile phone data, the nodes usually represent people or geographical locations, and the links indicate the occurrence of communication, such as a call being placed, or an SMS being sent. A particular feature of mobile phone networks is the natural emergence of a community structure [16]. Within a network, communities are groups of nodes whose internal connections are stronger or denser than those that link nodes in different groups. Many synthetic models and real-world complex systems have a modular structure, whose function and effects on the static and dynamical properties of the system have been extensively studied [17–31]. Techniques for analyzing communities in evolving networks have also been studied in the literature [32]. Previous work has focused on the evolution of communities in networks derived from mobile phone records to investigate the robustness of a person’s social signature [33] or to study the different dynamics of small and large groups [34]. Voting patterns in the United States Senate have also been investigated within the framework of evolving communities, with the aim of capturing both individual and group trends across time [35].

Here, we analyse the community structure of the network induced by mobile phone calls placed and received within the Milan metropolitan area, in northern Italy, over a period of two months, revealing the spatial and temporal patterns in the local communications. We aim to investigate whether communities in a mobile phone network reflect the patterns of our daily lives and behaviour, and whether they carry a signature of socially relevant events. After describing the data sets used in the analysis, we show how communities vary over a single day, a week, and several weeks.

Existing work has investigated how circadian and weekly patterns affect our communications mostly at the individual level. E-mail communication patterns, such as heavy tails in the temporal distribution of consecutive e-mails, can be accurately reproduced incorporating circadian and weekly cycles in the models [36]. Similarly, mobile phone communications also exibhit a heavy tail behaviour in the distribution of times between calls that can be explained by a combination of our circadian and weekly patterns as well as our task-execution behaviour [37]. Geographical information about mobile phone communications can also provide valuable insights on our behaviour and mobility [38]. Individual differences in patterns of phone calls have also been investigated using a combination of mobile communication and questionnaire data, showing that these differences are not only due to circadian rhythms but also reflect our social behaviour [39]. Our analysis shows how analogous patterns are present not only at the individual level, but also at the community level in networks induced by mobile phone communications.

## 2 Preliminary analysis

**Fig 1:**
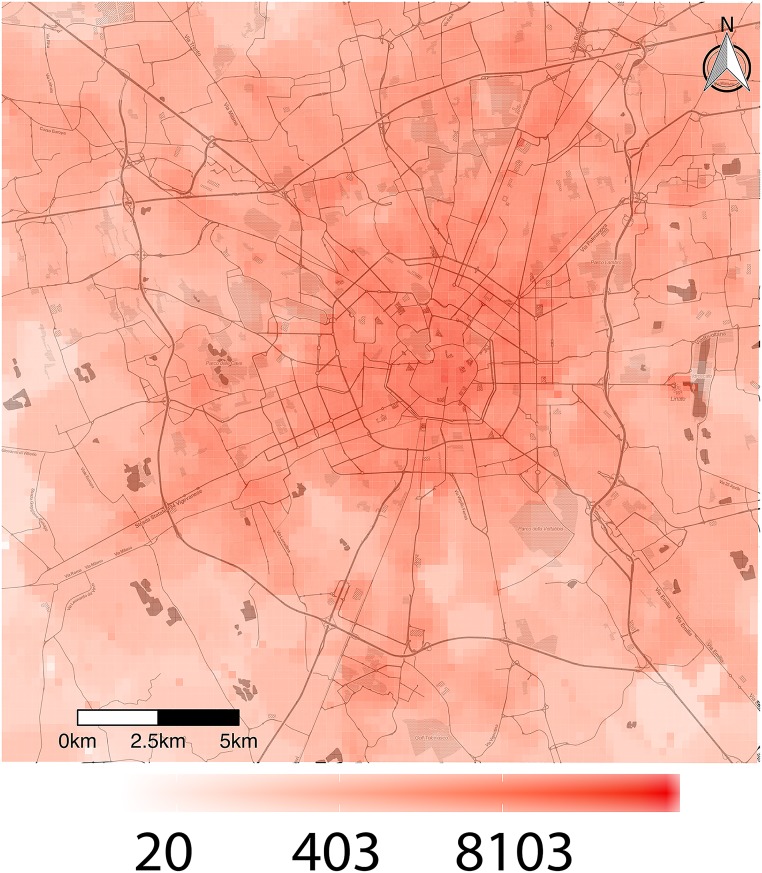
Radially decreasing mobile phone activity, as defined in Eq 1. The activities of the cells are highest in downtown Milan, and roughly decrease with distance from the city centre. Notable exceptions are the airport and residential suburbs. This map was generated with data from *OpenStreetMap* (OpenStreetMap contributors [42]) and tiles from *Stamen Design* [43].

The dataset we retrieved contains the anonymized records of phone calls between geographical areas in the city of Milan and surroundings, as presented in Fig 1. Before making them available, the mobile phone provider aggregated the data both spatially into a grid with 10000 cells, each cell being square of side 235m, and also temporally at a ten minute granularity. The area of analysis is presented in Fig 1. All data sets used were released as part of *Telecom Italia Big Data Challenge 2014*, and are publicly available at [40]. The period of analysis goes from 1 November 2013 to 31 December 2013. A more detailed description of how the dataset was constructed is presented in [41]. We study the cell activity by constructing a series of weighted networks. The nodes in these networks represent geographical locations, and the link strength is proportional to the volume of calls between the corresponding cells. The volume of calls is given by the mobile phone provider, and is proportional to the number of phone calls between cells. For privacy reasons, the proportionality constant is only known to the provider.

### 2.1 Network construction

For a preliminary characterization of the networks structure, we build a single network aggregating all time intervals, which we refer to as the aggregate. As the whole period of analysis consists of 8784 time intervals, the edge weights are defined as:

$$\begin{array}{c} \omega_{ij}=\frac{1}{Z}\left(\sum_{t=1}^{8784}{\bar{w}}_{ij}+\sum_{t=1}^{8784}{\bar{w}}_{ji}\right)\;. \end{array}$$ (1)

In the equation above, ${\bar{w}}_{ij}$ is the volume of calls originating on node *i* and reaching node *j*. Thus, the edge weight *ω*_*ij*_ is the normalized volume of phone calls between nodes *i* and *j*. The normalization constant $Z=\max_{i,j}\{\sum_{t=1}^{8784}{\bar{w}}_{ij}+\sum_{t=1}^{8784}{\bar{w}}_{ji}\}$ is chosen so that the strongest edge weight is 1. With these definitions, we assign to each node *i* an activity *k*, defined as:

$$\begin{array}{c} k_{i}=\sum_{j=1}^{10,000}\omega_{ij}\;. \end{array}$$

The activity is a weighted equivalent of the node degree, measuring the total strength of all the connections involving a given node. A geographical heat map of the activities, in the right panel of Fig 1, shows that a higher call volume is recorded in downtown Milan, in agreement with the intuitive notion that the centre is the busiest part of the territory.

### 2.2 Community detection algorithm

To analyze the network thus created, we use the community detection algorithm described in Ref. [44]. This is a recent fast spectral method that uses several refinement steps to identify the network partition that tries to maximise the modularity

$$\begin{array}{c} q=\frac{1}{2m}\sum_{ij}\left(A_{ij}-\frac{d_{i}d_{j}}{2m}\right)\delta_{c_{i},c_{j}}\;. \end{array}$$

and that has been shown to produce the highest values of modularity on several benchmark networks, when compared to other available algorithms. In the equation above, the sum runs over all pairs of nodes, *m* is the total number of edges in the network, *d*_*i*_ is the degree of node *i*, *c*_*i*_ is the community to which node *i* is assigned, *δ* is Kronecker’s symbol, and *A* is the adjacency matrix, whose (*i*, *j*) element is 1 if there is an edge between nodes *i* and *j*, and 0 otherwise. The values of modularity are constrained between −1 and 1, with higher values corresponding to better partitions. The algorithm also provides the effect size of the detected partition in terms of a *z*-score, which is the number of standard deviations that separate the measured modularity from that of an Erdős-Rényi null model, as fully detailed in Ref. [44].

### 2.3 Thresholding of the network

**Fig 2:**
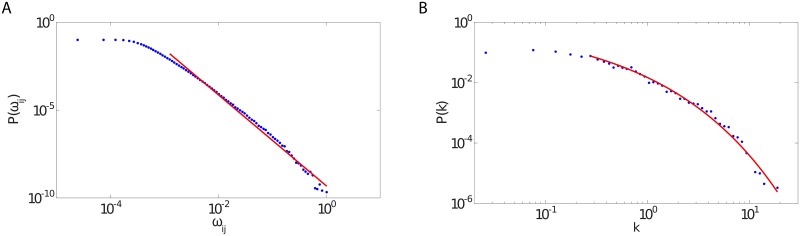
Weights and activities of the aggregate network. The distribution of the edge weights in the temporally aggregated network (left panel) shows a slow decay, with a tail that is well fitted by a power-law with exponent −2.59. The activities (right panel) follow instead a stretched exponential (Eq 2), with k* = 0.023 and *α* = 0.383. The values of *τ* used in the analysis are: (1 × 10^−6^, 5 × 10^−6^, 1 × 10^−5^, 5 × 10^−5^, 1 × 10^−4^, 5 × 10^−4^, 1 × 10^−3^, 2.5 × 10^−3^, 5 × 10^−3^, 7.5 × 10^−3^, 1 × 10^−2^, 2.5 × 10^−2^, 5 × 10^−2^).

**Fig 3:**
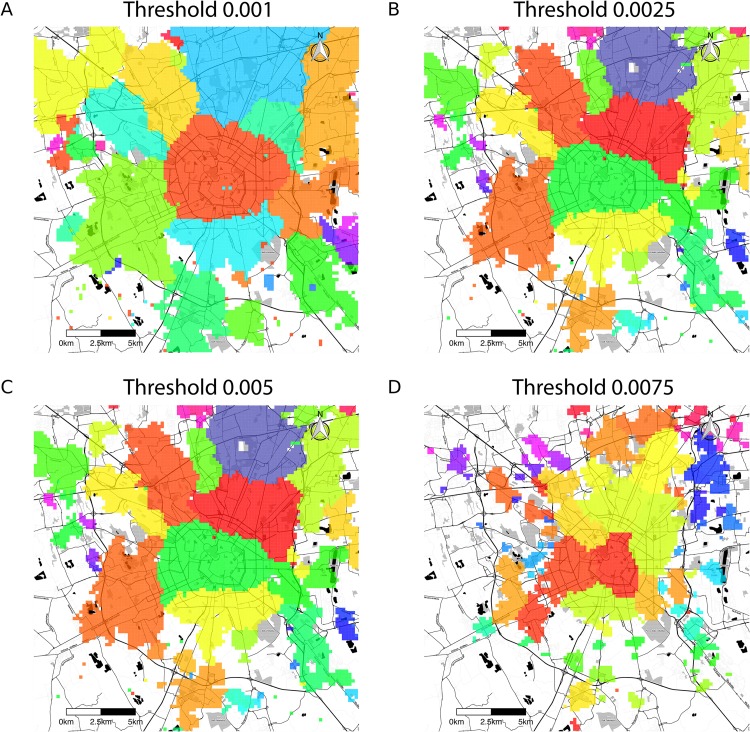
Hierarchical backbone of communication communities. For low values of the threshold *τ* the noise still dominates the community structure detected. However, after the critical threshold of 0.005, increasing *τ* only causes the communities to fragment into sub-modules. Areas left uncoloured correspond to isolated nodes in the thresholded network. These maps were generated with data from *OpenStreetMap* (OpenStreetMap contributors [42]) and tiles from *Stamen Design* [43].

The study of the community structure could be performed, in principle, on the weighted network. However, such analysis could be sensitive to the presence of noise, i.e., very weak links that may mask the underlying structural character of the network. This is a particularly likely occurrence, given the slow-tail decay in the distributions of weights and activities (Fig 2), which makes the weakest edge strength and the lowest node activity the most probable. More precisely, the distribution of weights exhibits a power-law tail with exponent −2.59, while the activity distribution follows a clear stretched exponential

$$\begin{array}{c} P\left(k\right)∼e^{-{\left(\frac{k}{k^{*}}\right)}^{\alpha }}\;, \end{array}$$ (2)

with k* = 0.023 and *α* = 0.383. Thus, we prefer to *threshold* the aggregate by introducing a parameter *τ*: for any chosen value of *τ*, we create a network by removing from the aggregate any edge whose weight is less than *τ*, and considering all other edges as unweighted. This ensures that we remove all weak links that may alter the underlying topology of the network. We run the algorithm 100 times on each thresholded network, and select the partition with the highest value of modularity. As the values of *τ* increase, the number of nodes *N* and that of edges *m* in the network decrease. In particular, for the cases reported in Fig 3, we have:

$$\begin{array}{c} \tau =0.001,N=7204,m=538976\\ \tau =0.0025,N=5701,m=186853\\ \tau =0.005,N=4266,m=76086\\ \tau =0.0075,N=3455,m=43234 \end{array}$$

**Fig 4:**
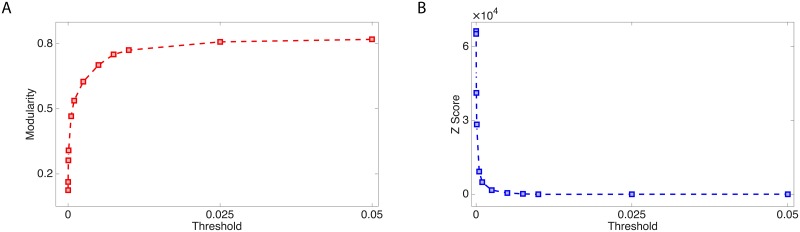
Threshold evolution of network modularity. For increasing values of the threshold, the modularity increases (panel A), apparently saturating at a value just above 0.8. For the same thresholds, the *z*-score, which is a measure of the effect size of a given modularity measurement, has a fast decay, indicating that the community structure quickly becomes similar to what would be found in a random network as more links are erased. The lines are guides for the eye.

We also note that the evolution of the detected community structure undergoes a significant change when *τ* reaches a “critical value” τ* ≈ 0.005. At lower thresholds, the communities change significantly with *τ*. Conversely, thresholds greater than τ* only result in fragmentation of the existing communities into smaller ones almost entirely contained within the parent module, without drastic changes in the overall structure. In addition, the individual communities correspond to connected areas of territory (Fig 3). A second effect we note is that increasing thresholds correspond at the same time to higher values of the modularity, and lower *z*-scores (Fig 4). Explaining this behaviour in detail is a complex problem, since, to a preliminary investigation, it appears to depend on the distribution of weights between modules, and it will be addressed in future publications. A preliminary understanding of this may come from the fact that weak links are more likely to connect different communities. Removing these links would therefore enhance the community structure and result in an increase in the modularity value.

For the analysis of our data, we choose to work on the network corresponding to the critical threshold, as this provides a good balance between two necessities, namely that of a large enough threshold to remove the noise that might mask the community structure, and that of a small enough threshold to avoid excessive fragmentation. Even though this choice is arbitrary, our results are robust with respect to small threshold variations. Also, we show below that analogous results hold for weighted networks where we keep all edge weights unchanged. Thus, to take advantage of faster computational times, we use the unweighted network for further analysis.

## 3 Time evolution of communities

Our first goal is to to investigate the communication patterns that appear over time at a community level, to gain insights in the emergent structures of human communication. We start by studying how the communities evolve on the time scale of single days. To do so, we create an aggregate network for each day over the period covered by our data, and perform community detection on each of them as described above, with the aim of quantifying the difference between the community structures in the different “daily” networks. One of the most widely used methods for the actual comparison and evaluation of such differences is to calculate the *Normalised Mutual Information* (NMI), a measure borrowed from information theory [45–51]. To find the NMI between two partitions *C* and $\tilde{C}$, first treat them as random variables and compute their mutual information:

$$\begin{array}{c} I\left(C,\tilde{C}\right)=\sum_{i=1}^{n_{C}}\sum_{j=1}^{n_{\tilde{C}}}\frac{V_{ij}}{N}\log\left(\frac{V_{ij}N}{V_{i}V_{j}}\right)\;, \end{array}$$

where the *V*_*ij*_ are the elements of the *confusion matrix* *V*, whose entries are the numbers of nodes belonging to community *i* in partition *C* and to community *j* in partition $\tilde{C}$, *V*_*i*_ denotes the sum over the elements of row *i* in *V*, and *N* is the total number of nodes. Then the NMI between two partitions is defined as

$$\begin{array}{ccc} NMI(C,\tilde{C}) & = & \frac{-2I(C,\tilde{C})}{\sum_{i=1}^{n_{C}}\frac{V_{i}}{N}\log\frac{V_{i}}{N}+\sum_{j=1}^{n_{\tilde{C}}}\frac{V_{j}}{N}\log\frac{V_{j}}{N}}\\ & = & \frac{-2\sum_{i=1}^{n_{C}}\sum_{j=1}^{n_{\tilde{C}}}V_{ij}\log\left(\frac{V_{ij}N}{V_{i}V_{j}}\right)}{\sum_{i=1}^{n_{C}}V_{i}\log\frac{V_{i}}{N}+\sum_{j=1}^{n_{\tilde{C}}}V_{j}\log\frac{V_{j}}{N}}\;. \end{array}$$

The normalised mutual information can assume values ranging from 0 to 1. High values indicate stronger similarity between the two partitions, with $NMI(C,\tilde{C})=1$ found if the two partitions are identical. Conversely, partitions that are totally independent from each other have a normalised mutual information of 0.

**Fig 5:**
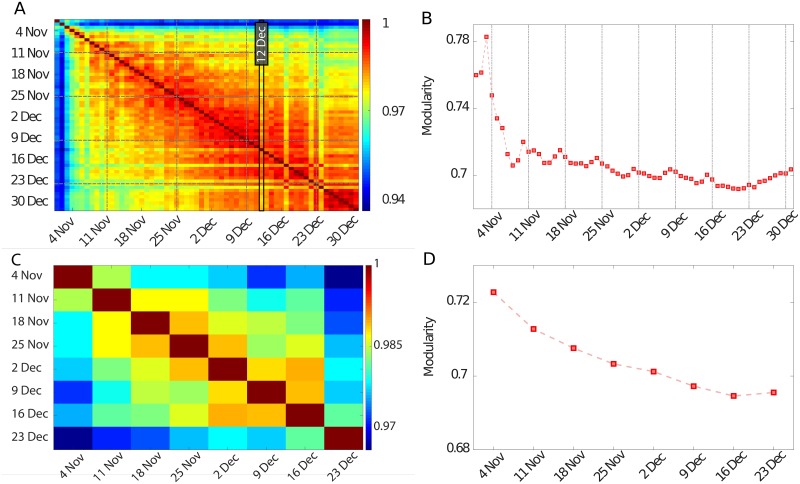
Determining the time-scale of social dynamics. Panel A depicts the Normalised Mutual Information between partitions at different days, showing a strong similarity between all communities during the two months analyzed. Panel B presents the evolution of modularity during the period of analysis. Vertical dashed lines correspond the the beginning of the working week (Monday). The modularity has an unusual spike in the first days of November, probably due to a bank holiday long weekend, but only oscillates around a constant value for subsequent periods. We note that the modularity on weekends is consistently higher than it was during the working days of the corresponding week. The NMI analysis of partitions corresponding to different weeks, in Panel C, shows a strong similarity between all communities. Panel D illustrates the evolution of modularity of the weekly networks, with labels indicating the first day of each week. In agreement with the previous analysis, the modularity has a higher value in the first week of November.

The NMI values we find are always quite high (Fig 5A), indicating a strong similarity in the community structure across different days. This provides evidence of the robustness of the structure of the mobile phone call network over the 24-hour time scale, with only minor changes between communities across the two months. Nonetheless, some days stand out as significantly different from the average. First, we observe an unusual structure in the first few days of November. This is most probably due to the particular nature of that period, which includes a bank holiday covering an important mandated Catholic holiday (1 November). In addition, in 2013, the holiday fell on a Friday, causing a “long weekend”. We also note that the community structure in these days had a substantially higher modularity than the average for the rest of the period (Fig 5B). Another remarkable difference in the structure appears on 12 December. This is likely caused by the combination of three major events happening in Milan on that day: 1) an annual demonstration in memory of the controversial *Piazza Fontana Bombing*, a terrorist attack that took place on 12 December 1969; 2) a second demonstration, part of ongoing protests against the Italian government; and 3) a major concert of One Direction, a highly popular pop boy band. Notably, both political demonstrations saw the occurrence of clashes between demonstrator and police forces, while the concert gathered thousands of people across the city for the whole day. The co-occurrence of these events clearly disrupted the usual patterns of communications in the city, causing the highly unusual community structure observed on that day. Finally, the changes in structure detected on 22 December and 24 December likely reflect the particular nature of this period of the year. In particular, 22 December was the last Sunday before Christmas, a day traditionally devoted to the final purchases before the start of the holiday period. Notice that these results provide direct evidence of how one can use mobile phone activity to extract information on people’s behaviour within social groups and directly detect socially relevant changes in their patterns. The data also allow us to infer a strong similarity in the last week of our analysis period, which corresponds to Christmas and New Year’s holidays. This supports the idea that communities in the communication networks closely reflect our behaviour. In the holiday period, people traditionally spend more time with their families, and reduce the frequency of contacts with acquaintances and other people outside their close-friend circles. Thus, the structure of communications is better defined, and links between different communities become less important, causing an increase in modularity. Also, this is an indication that the agents participating in communication tend to remain stable over this time period.

The analysis of the daily NMI also shows that days close to each other have a consistently higher similarity, suggesting that changes in the community structure happen over a longer time scale than just one day. To investigate this, we build aggregates for each entire week in the period of analysis and perform community detection as above. Our findings (Fig 5) show that weeks close to each other are very similar, and the NMI exhibits a slower decay than what we observed in the daily structure. This suggests that the variability in the structure is due to a slow dynamics of the communities happening over different days and repeating with the period of a week. In the next section, we present a detailed analysis of this two-time-scale behaviour. To verify the statistical significance of these results, we validated them against an appropriate null model. The results, confirming our findings, are detailed in S1 File in the *Supporting Information*.

## 4 Period analysis of network structure

To investigate the periodic behaviour of the communication patterns, we employ the same NMI comparison approach introduced in the previous section, by building aggregates for each different day of the week. In other words, we construct seven different networks, the first aggregating the data collected on all Mondays, the second with the data from all Tuesdays, and so on up to the seventh network which corresponds to all the Sundays. Then, we build a daily NMI matrix where each element is the NMI between the structures detected on the corresponding aggregates.

**Fig 6:**
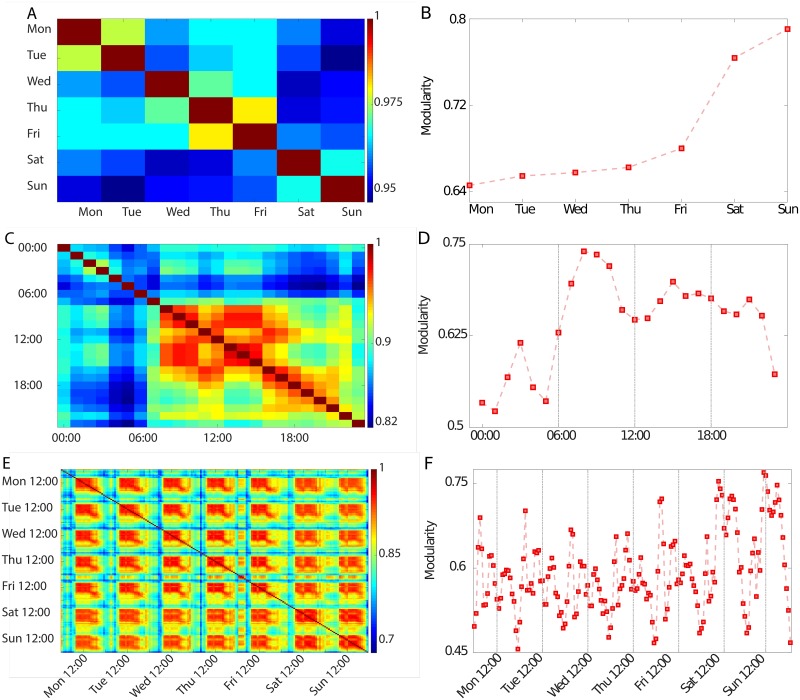
Weekly, daily, and hourly-weekly routines. Panels A, C and E show the Normalised Mutual Information between partitions of aggregates corresponding to different days, different hours, and different hours of each day, respectively. Communication communities on weekends are evidently different from those on working days. Also, waking hours are much more stable than the night, with two clear blocks corresponding to working hours and evening time. Moreover, the hourly-weekly analysis shows a striking structure corresponding to blocks of highly similar communities during the daytime. The modularities for the three types of networks (Panels B, D and F), show that communities are much tighter on weekends and during waking hours than they are on weekdays and during the night, with the exception of the weekend nights that are highly modular.

The results, in Fig 6A, show that different days are always very similar, with an NMI consistently greater than 0.95. However, a difference is still evident between working days and weekends, in agreement with the daily analysis. In fact, the NMI reaches its highest values when comparing either two working days or the two days of the weekend, while the smallest values are found when comparing a weekend day and a working day. This difference also corresponds to a higher value of modularity for weekend days than for the rest of the week (Fig 6B), supporting the idea that on non-working days people tend to be active only within their closest social circles. Note that these results illustrate the ease with which one can extract quantifiable information about the behaviour of people in social contexts from communication records, even if completely anonymized and already geographically aggregated in their raw form.

The results found so far show that we can clearly detect the difference in population behaviour over the different days of the week. However, human activities also change at the shorter time scale of hours. Thus, we investigate the changes in average community structure during a day by constructing 24 different networks, each aggregating the data collected during the same hour every day. For this analysis, we do not distinguish working days from weekends, and include all days available in our data set. The NMI matrix (Fig 6C) shows a remarkable difference between daily and nightly communities. The structure of communities at night does not present particular patterns, in agreement with the intuitive understanding that people only make sporadic and occasional calls during the night. We find blocks of high similarity during the day: a first block corresponds to highly similar communities during morning hours, covering roughly the first part of a working day; a second block can also be observed in the afternoon hours, when the second part of a working day happens. Finally, a last block extends over the evening hours. Working hours may result in stronger communities due to people having regular and repeated calls between offices of partner companies or fellow workers. We find these results remarkable, in that they confirm that mobile phone communications are closely related to human behaviour even at a community level. Fig 6D shows the evolution of modularity for the hourly networks. We find that the waking hours correspond in general to stronger communities, with modularity dips in correspondence of the periods traditionally linked to lunch (12:00–13:00) and dinner (20:00). We note that part of these differences could also be due to other global properties of the network that change during the day and that are likely to affect the community structure. For instance, the average fraction of links in daytime networks (8am to 11pm) is 0.88%, whereas during the night (11pm to 8am) it is 0.64%. However, while this difference may be one of the reasons of the observed change in community structure, the pattern observed in Fig 6D cannot be explained in terms of different density of the networks alone.

Finally, to clearly show the periodic nature of the network, we analyze the data differentiating for given hours *and* days of the week. We create 168 networks, each aggregating the data corresponding to the same hour and the same day of the week, and perform an NMI analysis. The results, in Fig 6E, show the emergence of a clear structure, where partitions obtained at daytime hours are strongly similar, and cluster in blocks with high values of NMI, separated by lower similarity partitions corresponding to the nights. Investigating this result more closely, we notice that higher similarities are observed between different daytime hours of the same day. The evolution of modularity (Fig 6F) displays again a similar pattern to the one previously observed with two peaks in the value of modularity in the morning and afternoon and a lower value during the night. However, we also find a peak in the middle of the night, particularly strong during weekends. This might reflect the fact that phone activity is naturally lower during the night. Thus, it is highly likely that someone placing a nighttime call will not call more than a few close contacts, and will not receive a call back from people other than the persons originally called. This results in strong communities and a high modularity. Similarly, we also find a higher modularity during weekends than over weekdays, consistently with the social dynamics outlined before. In addition to validating these findings against a null model (S1 File in the *Supporting Information*), we also test their robustness using the method proposed by Mucha *et al*. [35], obtaining results that support our methodology (details in S2 File in the *Supporting Information*).

## 5 Conclusions

In conclusion, we have presented a study of the community structure of a mobile phone call network and discussed its evolution over time, revealing the temporal patterns in local communications. Our findings suggest that information about people’s behaviour and their interactions can be extracted from the community structure of networks induced by communication records. In fact, our results provide direct evidence of how one can use mobile phone activity to point out the occurrence of socially relevant events. The ease with which our method can be applied, coupled to the high granularity of the data available to telecommunication companies, suggests that it may be useful even as a real-time tool to detect the occurrence of such events or activities, as evidenced by our results related to the day of 12 December.

Our analysis also presents some limitations. The geographical and temporal aggregation of the data set may affect the network structure and pose challenges for the geographical interpretation of communities. A more refined analysis should investigate the detailed effects of the spatial aggregation, and in particular try to find an optimal level of aggregation that improves the granularity whilst preserving the privacy of the users. One other aspect worth of investigation is the relation between the community and the urban geography of the city, which we aim to address in future publications, as we believe that mobile phone providers, as well as authorities, have a strong interest in knowing which parts of a city communicate more strongly with others, and how these regions change over time. Another important consideration concerns the source of our data set, which is not the only provider in Italy, despite being quite prominent. This could, in principle, introduce biases in the analysis, even though we do not believe that the demographics of the users vary enough between providers to produce such effects. Having access to data from all mobile phone providers for this location could nonetheless allow one to perform a more complete analysis. These data could be integrated and represented as a multilayer network, whose communities could spread across different providers. However, mobile phone data are privately owned and quite difficult to access. Thus, we have focused on a unique but very detailed data set coming from the most popular mobile phone provider in Italy.

Finally, our work has also shown that circadian and weekly patterns can be found in mobile communications not only at the individual level, but also at the level of the community structure in the network of mobile phone calls. Future work should focus on the spatial nature of these cycles to assess how the geographical area underlying each community varies during a day or a week. Moreover, a model able to reproduce these patterns should also be investigated, in order to provide a better understanding of the mechanisms responsible for the observed patterns.

## Supporting information

S1 File Null model validation. Fig A. Validation of NMI analyses. Randomized NMI matrices for the daily (panel A), weekly (panel B), week aggregates (panel C), hourly (panel D) and hourly-weekly (panel E) show values that are roughly constant across the matrix, and always smaller than those observed in the original data. Also, we do not observe the patterns characterizing the NMI matrix presented in the main text, such as the separation between working days and weekends and the strong similarity between daytime communities. Times are reported in Central European Time (CET).
(PDF)

S2 File Weighted and multiplex analysis. Fig A. Weekly community structure NMI using multiplex detection. We observe results strongly similar to the results presented in the main text both with no coupling (Panel A) and with coupling between each node and its copies in the neighbouring layer (Panel B). The multiplex modularity value in the two cases is 0.6935 (*ω* = 0) and 0.6960 (*ω* = 0.1). These are compatible with the average value of modularity across the seven networks presented in the main text, which is 0.6934, thus compatible with this result. Fig B. Weekly community structure NMI using weighted multiplex detection. We observe results similar to the results presented in the main text. Here, we can also notice a smaller differentiation between weekday groups.
(PDF)
