# Supplementary material for: Analysis of the communities of an urban mobile phone network
Source: PLoS One. 2017 Mar 23;12(3):e0174198. doi: 10.1371/journal.pone.0174198 (PMC5363903; doi:10.1371/journal.pone.0174198)

To validate the NMI analyses, we build a null model by fixing number and size of the communities detected in each instance, and randomising the community labels assigned to each node. We compute the NMI matrix averaged over 100 randomizations, and compare it with the one presented in the main text. This allows us to verify whether our results can be attributed to randomness, or they represent an effect present in the data. The results, shown below, bear no resemblance to those in the main text. Also, in all null models we observe that the patterns characterizing the NMI matrices of the original data are absent. This shows that it is very highly unlikely that the structures detected arose due to random fluctuations.

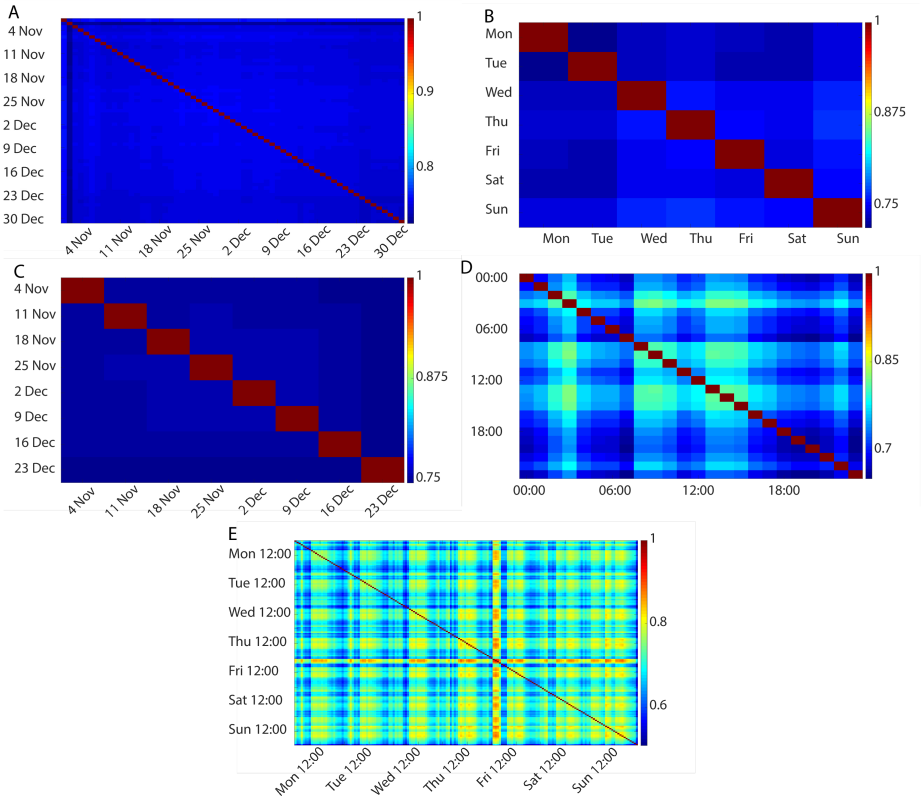

Supplement: S1 File — Fig A. Validation of NMI analyses. Randomized NMI matrices for the daily (panel A), weekly (panel B), week aggregates (panel C), hourly (panel D) and hourly-weekly (panel E) show values that are roughly constant across the matrix, and always smaller than those observed in the original data. Also, we do not observe the patterns characterizing the NMI matrix presented in the main text, such as the separation between working days and weekends and the strong similarity between daytime communities. Times are reported in Central European Time (CET). (PDF) [file pone.0174198.s001.pdf]
