# Supplementary material for: Analysis of the communities of an urban mobile phone network
Source: PLoS One. 2017 Mar 23;12(3):e0174198. doi: 10.1371/journal.pone.0174198 (PMC5363903; doi:10.1371/journal.pone.0174198)
Supplement: S2 File — Fig A. Weekly community structure NMI using multiplex detection. We observe results strongly similar to the results presented in the main text both with no coupling (Panel A) and with coupling between each node and its copies in the neighbouring layer (Panel B). The multiplex modularity value in the two cases is 0.6935 (ω = 0) and 0.6960 (ω = 0.1). These are compatible with the average value of modularity across the seven networks presented in the main text, which is 0.6934, thus compatible with this result. Fig B. Weekly community structure NMI using weighted multiplex detection. We observe results similar to the results presented in the main text. Here, we can also notice a smaller differentiation between weekday groups. (PDF) [file pone.0174198.s002.pdf]

To test the robustness of the results presented in the main text, we use the approach described in [1]. This method provides a generalisation of the classical modularity to the case of time-dependent and multiplex networks:

$$Q_m = \frac{1}{2\mu} \sum_{ijsr} \left[\left(A_{ijs} - \frac{k_{is}k_{js}}{2m_s} \right) \delta_{sr} + \omega \delta_{ij} \right] \delta_{g_{is},g_{jr}}$$

where the indices i and j refer to nodes, the indices s and r refer to layers, ω is a parameter that determines the strength of the coupling of a node to its copies in the neighbouring layers, μ is the sum of all the strengths across all layers and g_{is} is the community of node i in layer s. This quality function is then maximised using a generalisation of the Louvain method [2]. Notice that when $\omega = 0$, the layers are independent. Conversely, high values of ω increase the coupling to the point that all the replicas of each node are treated identically. This causes the communities found to be the same across layers, effectively neglecting the multiplex nature of the network. Thus, for this type of analysis, one needs to find an intermediate value of ω that offers a compromise between the two extremes. In our case, we choose $\omega = 0.1$, as the value above which the differences between layers start to smoothen. We consider the same thresholded networks used in the main text for each day of the week and assign a different layer to each of them, thus creating a multiplex network with 7 different layers. Fig A shows the NMI analysis of the partitions of the individual layers for $\omega = 0$ and $\omega = 0.1$. In both cases, we obtain results that are qualitatively similar to those presented in the main text. Moreover, the multiplex modularity Q_m in the two cases is 0.6935 and 0.6960, respectively, in agreement with the average modularity of the seven networks presented in the main text, which is 0.6934. Studying the effect of preserving the link weights is also of great interest for

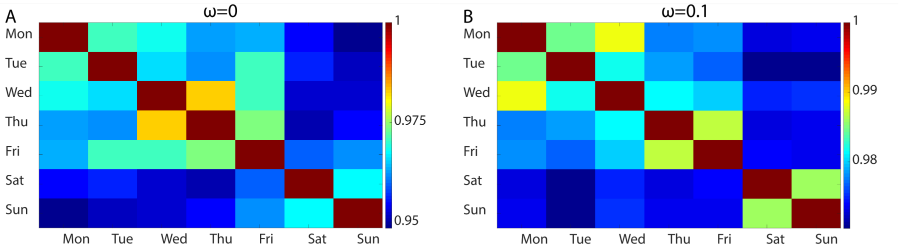

the application of this methodology. In the main text, we used a threshold parameter to remove weak links that may act as noise and mask the community structure. Here, we build aggregated networks for each day of the week keeping all links with their weights and analyze them with the method described above. As before, each layer in the multiplex corresponds to an aggregate network of a given day. Panel A in Fig B shows that if we preserve all the links with their original weights and leave the layers uncoupled, the difference between weekdays and weekends is not remarkable. Panel B in Fig B depicts the results when the coupling between the layers is $\omega = 0.1$. As before, we observe a structure

similar to the one presented in the main text, with the exception of a difference in the typical Friday communities.

This last analysis supports our hypothesis that thresholding removes the noise in the network and allows us to uncover the underlying community structure, while leaving the relevant structural properties unchanged. We also see that, if we want to keep all the links with their weights, a coupling between the layers is essential. However, the size of the multiplex grows really quickly when considering several layers, such as the hourly-weekly routine, where we would have 168 networks with 10000 nodes each. The multiplex could possible be even larger, depending on the granularity of the data, making its analysis not always feasible. Our methodology, instead, obtains valid results while analysing the networks separately, thus being faster and demanding less resources.

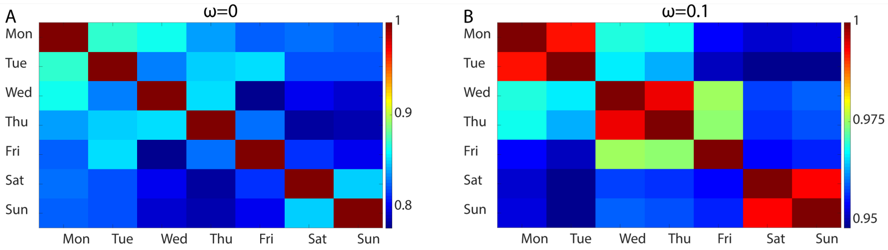

References

[1] Mucha, PJ, Richardson, T, Macon, K, Porter, MA & Onnela, JP. Community structure in time-dependent, multiscale, and multiplex networks. *Science*. 2010; **328**: 876–878

[2] Blondel, VD, Guillaume, JL, Lambiotte, R & Lefebvre, E. Fast unfolding of communities in large networks. *J. Stat. Mech. - Theory E.* 2008; P10008
